# Plasmonic Mesoporous Gold‐Based SERS Biosensor for Ovarian Cancer‐Derived Extracellular Vesicles

**DOI:** 10.1002/smll.202401817

**Published:** 2025-07-09

**Authors:** Javeria Bashir, Mostafa Kamal Masud, Asep Sugih Nugraha, Chia‐Hung Liu, Arya Vasanth, Aditya Ashok, S M Azad Hossain, Emtiaz Ahmed, Tanja Pejovic, Terry Morgan, John Hooper, Andrew Lai, Dominic Guanzon, Yusuf Valentino Kaneti, Md Shahriar A. Hossain, Carlos Salomon, Yusuke Yamauchi

**Affiliations:** ^1^ School of Mechanical and Mining Engineering Faculty of Engineering Architecture, and Information Technology (EAIT) The University of Queensland Brisbane Queensland 4072 Australia; ^2^ Australian Institute for Bioengineering and Nanotechnology (AIBN) The University of Queensland Brisbane Queensland 4072 Australia; ^3^ TMU Research Center of Urology and Kidney and Department of Urology School of Medicine College of Medicine Taipei Medical University 250 Wu‐Hsing Street Taipei 11031 Taiwan; ^4^ Department of Urology Shuang Ho Hospital Taipei Medical University 291 Zhongzheng Road, Zhonghe District New Taipei City 23561 Taiwan; ^5^ Department of Obstetrics and Gynecology Oregon Health & Science University Portland OR 97239‐3098 USA; ^6^ Department of Pathology Oregon Health & Science University Portland OR 97239‐3098 USA; ^7^ Mater Research Institute The University of Queensland South Brisbane Queensland 4101 Australia; ^8^ Translational Extracellular Vesicles in Obstetrics and Gynae‐Oncology Group UQ Centre for Clinical Research (UQCCR) Faculty of Medicine, and UQ Centre for Extracellular Vesicle Nanomedicine The University of Queensland Brisbane Queensland 4029 Australia; ^9^ Department of Materials Process Engineering Graduate School of Engineering Nagoya University Nagoya 464‐8603 Japan; ^10^ Department of Chemical and Biomolecular Engineering Yonsei University 50 Yonseiro, Seodaemun‐gu Seoul 03722 Republic of Korea

**Keywords:** extracellular vesicles, liquid biopsy, mesoporous gold, ovarian cancer, surface‐Enhanced Raman Spectroscopy (SERS)

## Abstract

Extracellular vesicles (EVs) are lipids bilayer‐delimited particles carrying bioactive molecules such as proteins, lipids, and nucleic acids, reflecting the physiological state of their origin. Found in biofluids like saliva, urine, blood, and peritoneal fluid, EVs serve as promising minimally invasive biomarkers for several conditions including cancer. However, achieving high sensitivity and specificity in EV detection remains technically challenging. Placental Alkaline Phosphatase (PLAP), an enzyme primarily expressed in the placenta during pregnancy, has emerged as a clinically relevant biomarker in gynecological malignancies, including ovarian cancer (OC). In this study, a nanoengineered mesoporous gold (mAu)‐based Surface‐Enhanced Raman Spectroscopy (SERS) platform is reported for the rapid and ultrasensitive detection of PLAP‐positive EVs in OC patients. The mAu offers high surface roughness, enabling numerous localized plasmonic hotspots that amplify Raman signals and improve probe and antibody loading. This allowed the detection of as few as 100 EVs mL^−1^ with excellent reproducibility (RSD < 5%,n = 3). In clinical validation (n = 30), the assay achieved 90% sensitivity (*95% CI: 60%–100%)* and 85% specificity (*95% CI: 15%–100%*) in distinguishing OC patients from those with benign and healthy controls, demonstrating superior performance compared to CA‐125. The mAu‐SERS platform shows considerable promise as a minimally invasive and clinically applicable diagnostic strategy for OC, especially for differential diagnosis for their ability to distingluis between benign and OC conditions.

## Graphical abstract

The nanoengineered mesoporous gold (mAu)‐based SERS platform enables rapid, sensitive detection of PLAP‐positive extracellular vesicles (EVs) in ovarian cancer (OC), with detection limits as low as 100 EVs mL^−1^. Achieving over 80% sensitivity and 98% specificity in clinical samples, this cost‐effective, non‐invasive tool shows promise for early cancer detection and population‐wide screening.

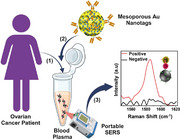

## 1 Introduction

Ovarian cancer (OC) is one of the leading causes of gynecological cancer deaths and is ranked as the eighth most common malignancy among women worldwide.^[^ ^1^ ^]^ The lack of effective screening methods to detect asymptomatic women with OC, particularly in stages I or II, leads to late‐stage diagnoses and significantly compromises patient outcomes.^[^ ^2^ ^]^ Current strategies for early detection, treatment, and monitoring of treatment response have had limited success in improving survival rates and quality of life for OC patients.^[^ ^3^ ^]^ Tumor progression depends on cell‐to‐cell communication, enabling cancer cells to reprogram near and distant cells within the tumor microenvironment.^[^ ^4^ ^]^ Recent studies have demonstrated the important role of extracellular vesicles (EVs) in facilitating bidirectional communication between normal and cancerous cells.^[^ ^5^, ^6^, ^7^ ^]^ Released by a range of cells, including tumor cells, EVs are enclosed by a protective lipid bilayer and contain essential biomolecules such as DNA, RNA (including messenger RNA and microRNA), and proteins. These vesicles play a crucial role in mediating signaling pathways that are critical to OC metastasis and disease progression.^[^ ^8^, ^9^ ^]^ Hence, the investigation of EVs offers a promising avenue for advancing detection, treatment, and prognostic strategies for OC.

The early detection of OC remains a significant clinical challenge and an essential goal in improving patient outcomes. Advancements in early detection methods could provide valuable insights into how early diagnosis affects disease progression and survival rates.^[^ ^10^, ^11^ ^]^ In particular, progress in technologies for isolating and characterizing EVs, (e.g., nanoparticle‐tracking analysis‐NTA and next‐generation sequencing), is anticipated to significantly enhance the reliability and efficiency of EV‐based diagnostics.^[^ ^12^ ^]^ EVs hold tremendous potential in the field of diagnostics, as they can be isolated from various biofluids such as blood, plasma, serum, urine, and saliva. They can serve as biomarkers for the detection and monitoring of cancer.^[^ ^13^ ^]^ Bioactive molecules like nucleic acids, lipids and proteins, contain EVs, which can provide critical information about the characteristics of OC cells.^[^ ^14^ ^]^ Analyzing these specific molecules, novel non‐invasive diagnostic techniques can be developed, offering a cost‐effective and less invasive alternative to traditional diagnostic techniques.^[^ ^15^, ^16^ ^]^

Several innovative approaches have been developed for EV detection, including fluorescence‐integrated microfluidics, electrochemistry, and field‐effect transistors.^[^ ^17^ ^]^ While these methods have shown considerable promise, they often require sophisticated equipment and complex procedures, greatly limiting their practical applications. Therefore, it is crucial to establish a highly sensitive technique for detecting EVs.^[^ ^18^, ^19^ ^]^ In this regard, Surface‐Enhanced Raman Scattering (SERS) has emerged as a superior alternative to these conventional methods. SERS significantly enhances signal intensity and sensitivity, allowing for the detection of biomarkers at the molecular level, even at extremely low concentrations.^[^ ^20^, ^21^ ^]^ Its high selectivity enables accurate analysis in complex biological samples. In addition, SERS allows for real‐time monitoring of biomarker dynamics by providing rapid detection. Given these unique advantages, SERS holds great potential in the field of disease diagnostics and biomedical research, particularly for detecting EVs. SERS‐based bioassays primarily rely on nanostructures made from plasmonic noble metals such as gold (Au) or silver (Ag).^[^ ^22^ ^]^ Au, in particular, can generate SERS signals owing to its strong interaction with electromagnetic (EM) radiation in the visible spectrum. Moreover, its biocompatibility makes it especially valuable for biomedical applications.^[^ ^23^ ^]^ Variations in shapes and surface properties of nanostructures affect the distribution and amplitude of the EM field, while size‐dependent plasmonic properties and the arrangement of nanostructures generate hotspots, resulting in amplified Raman signals.^[^ ^24^ ^]^ A previous study has shown that combining Au, Ag, and Cu into trimetallic alloys results in a significant boost in the SERS response due to their unique plasmonic properties.^[^ ^25^ ^]^ However, the synthesis of such multi‐metallic alloys is complex, requiring intricate chemistry, and often leads to inconsistencies in results. Hence, developing single nanoparticles (NPs) with exceptional SERS signal enhancement capabilities is highly desirable. In this context, mesoporous gold (mAu) presents significant advantages for preparing SERS reporters. The precisely engineered exterior surface and interior voids of mAu provide large surface areas and spacious cavities, which enhance their physicochemical properties. The exposed high‐index facets of Au further enhance the activity and selectivity of mAu for enhancing SERS signals. The pores within mAu impose the creation of highly concentrated and complex EM fields through surface plasmon resonances.^[^ ^26^ ^]^ As a result, mAu plays an important role in amplifying SERS signals, enabling ultrasensitive detection of disease‐specific EVs. When incident light interacts with Au nanostructures, it stimulates the generation of localized surface plasmon resonances (LSPRs), which significantly enhance the EM field near the rough surfaces and pores. To achieve high sensitivity and specificity in SERS‐based EV detection assays, it is vital to design mAu particles with optimized shapes, sizes, and surface properties.

Placental alkaline phosphatase (PLAP) is a glycoprotein enzyme typically found in high levels in the placenta and EVs during pregnancy.^[^ ^27^, ^28^ ^]^ While PLAP is primarily associated with normal placental tissue, elevated levels of PLAP have been observed in certain types of OC, particularly germ cell tumors and dysgerminomas.^[^ ^29^, ^30^, ^31^ ^]^ While the exact role of PLAP in OC remains unclear, it is believed to contribute to tumor growth and progression. Elevated PLAP levels in OC patients have been linked to more aggressive disease and a poorer prognosis^.[^ ^32^ ^]^ Additionally, PLAP has been suggested as a potential biomarker for monitoring treatment response and disease recurrence in OC patients.

In this study, we demonstrate the enhanced SERS performance of mAu NPs over their non‐porous counterparts. We utilize mAu‐based nanotags to screen PLAP‐positive EVs (PLAP^+ve^ EVs) in a magnetic bead‐based sandwich assay, isolating them from plasma samples. The porous architecture of mAu provides an expansive surface area, significantly increasing the number of active sites and “hotspots” for superior detection sensitivity. The assay, performed in a simple Eppendorf tube, uses a handheld Raman spectrophotometer for SERS readout, showcasing its portability and practical application. Validation with clinical samples from OC patients demonstrates the assay's remarkable ability to differentiate between cancer patients and healthy/benign controls, achieving a sensitivity of over 80% at a specificity of 98%. This innovative approach holds significant potential for minimally invasive cancer diagnostics.

## 2 Results and Discussion

### 2.1 Synthesis and Characterization of mAu Particles

**Figure 1:**
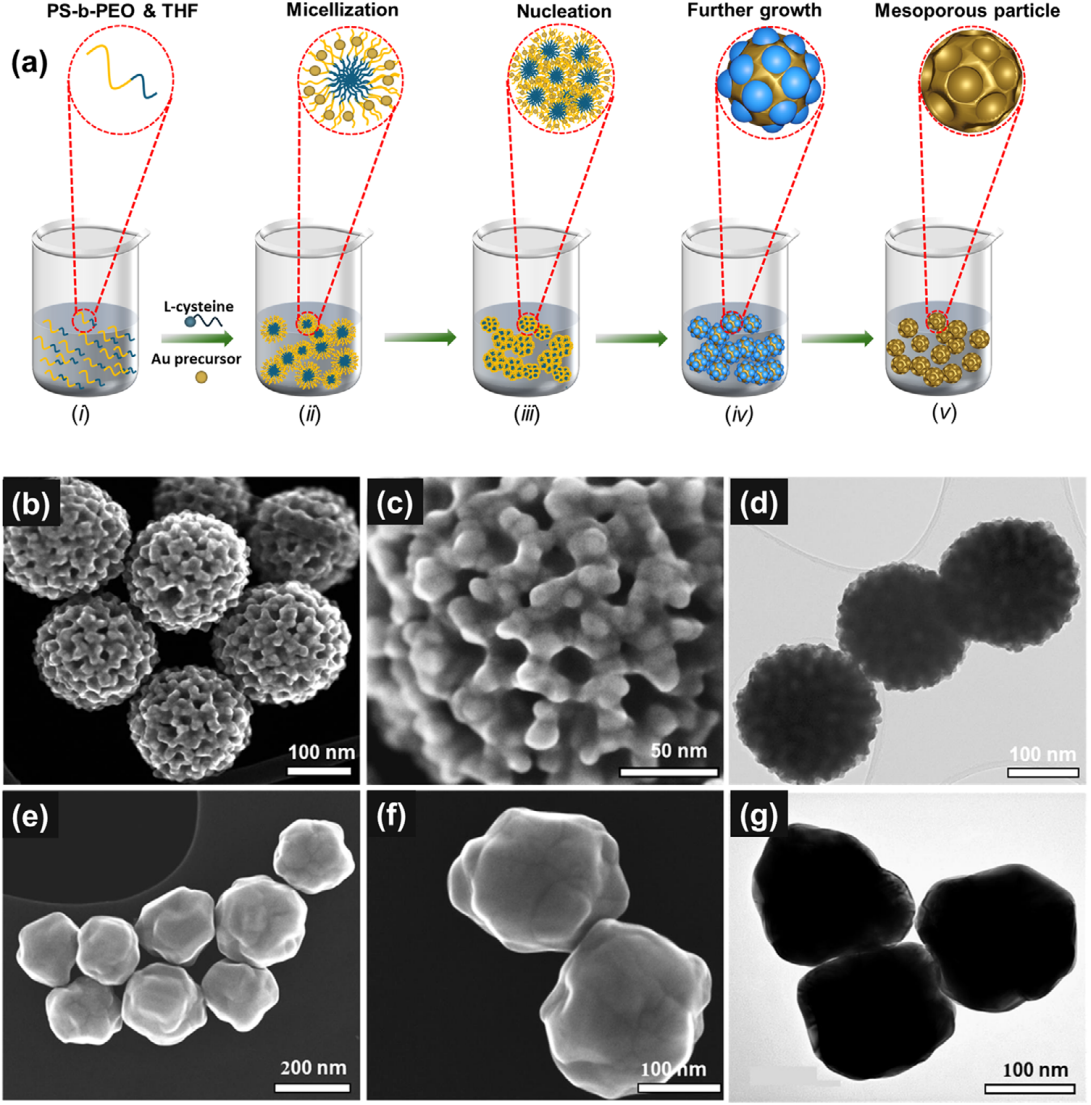
a) A schematic diagram depicting the synthesis of mAu NPs using block copolymer micelles (BCMs) as pore‐directing agents. Initiated by l‐cysteine and KOH, the Au precursor undergoes metallogelation and cluster formation, which are subsequently absorbed within the PEO shell of the BCMs (*i*,*ii*). This absorption induces the aggregation of BCMs (*i*), leading to the formation of Au metal‐containing micelles (*iv*). The reducing agent, ascorbic acid (AA), facilitates the reduction of Au present in the BCMs. Finally, the removal of BCMs generates the mAu NPs (*v*). b,c) SEM and d) TEM images of mAu NPs synthesized using PS_18000_‐*b*‐PEO_7500_ BCMs. e,f) SEM and g) TEM images of non‐porous Au NPs.

Plasmonic mAu NPs were synthesized using a soft‐templating approach. The typical process involved the formation of polymeric micelles by dissolving polystyrene‐*block*‐polyethylene oxide (PS‐*b*‐PEO) diblock copolymer in tetrahydrofuran (THF) (**Figure** **1a‐i–v**). Following this, ethanol was added as a co‐solvent, followed by the addition of KOH and L‐cysteine, resulting in a cloudy solution due to the formation of PS‐*b*‐PEO micelles (Figure 1a‐ii). Then, the Au precursor solution was introduced. Subsequently, a reducing agent was added, inducing a noticeable color change as metallic Au formed through reduction  (Figure 1–iii). The mixture was stirred for 15 minutes to ensure the chemical reduction process was fully completed (Figure 1a‐iv). Finally, the as prepared mAu NPs were isolated by employing washing and centrifugation processes to remove the block copolymer template (Figure 1a‐v). Non‐porous Au NPs with similar geometry were also synthesized to compare their SERS performance for biosensing.

**Figure 2:**
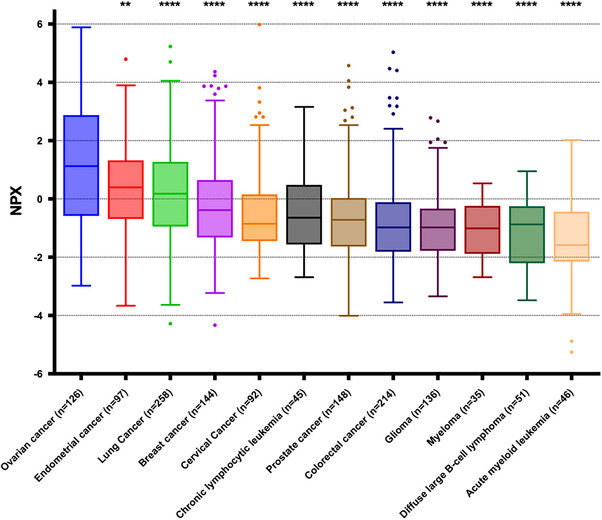
In‐silico analysis of PLAP specificity in OC using publicly available data from the Uppsala‐Umeå Comprehensive Cancer Consortium (U‐CAN) biobank, assessing expression across 13 different cancer types.

The structures of mAu and non‐porous Au NPs were analyzed using both scanning electron microscopy (SEM) and transmission electron microscopy (TEM), as shown in Figure 1b–g. The mAu NPs were imaged using SEM to determine their external dimensions. As depicted in Figure 1b–d, the resulting mAu exhibit a highly uniform spherical shape and size. From examinations of over 200 particles, the average diameter of this sample is determined to be ≈197.8 ± 5 nm, as seen in Figure S1a (Supporting Information). SEM imaging further reveals that these NPs exhibit uniform mesopores with an average pore size of ≈20 ± 1.3 nm (Figure S1b, Supporting Information). This uniformity in pore size signifies the successful formation of the intended mesoporous architecture. The TEM image in **Figure** **2**d further confirms the presence of uniform mesopores within these mAu NPs. Figure 1e–g displays the high‐resolution SEM and TEM images of the synthesized non‐porous Au NPs. These non‐porous Au NPs have almost the same size as the mAu NPs, but lack mesopores within the particles. The reduced XRD peak intensity and broadening observed for the mAu NPs in Figure S2 (Supporting Information) indicate a decrease in the crystallinity of Au, due to the formation of mesopores within the particles. HRTEM images of both mAu and non‐porous Au particles show no significant variation in d‐spacing, which measures 0.23 nm and is assignable to the (111) plane of face‐centered cubic (fcc) Au near the edges of both samples (Figure S3, Supporting Information).

### 2.2 Plasmonic Behavior of mAu NPs

Roughened Au surfaces with nanoscale features can enhance SERS signals.^[^ ^33^ ^]^ Similarly, mAu NPs with rough surfaces can create numerous local EM hotspots due to plasmonic effects, resulting in a higher enhancement of the Raman signal.^[^ ^34^ ^]^ The increased surface area and high EM enhancement contribute to this overall enhancement. Both mAu and non‐porous Au particles were functionalized with a SERS reporter 4‐MBA, and infrared absorbance spectra were obtained to identify the distinctive peaks of 4‐MBA (Figure S4a, Supporting Information). Both Au samples show distinctive SERS peaks corresponding to 4‐MBA; however, the mAu particles exhibit a significantly more intense absorbance peak compared to their non‐porous counterparts. Specifically, mAu particles show a two‐fold signal enhancement compared to their non‐porous counterparts (Figure S4b, Supporting Information), which can be attributed to the presence of hotspots resulting from the mesoporous architecture.

We have previously shown that the introduction of pores in the Au NPs modifies the plasmon resonance by altering its range and intensity through the coupling of dipolar and higher‐order plasmon modes.^[^ ^35^ ^]^ Large porosity promotes the formation of nanoscale junctions by creating subdivided regions within the particles, leading to an interconnected network with localized optical conductance. Additionally, large pores cause symmetry breaking by providing sufficient size of the particle to interact with time‐dependent electric fields. This facilitates the excitation of hybridized plasmon modes with asymmetric charge distributions, resulting in stronger local field enhancements. .^[^ ^36^ ^]^ This results in hotspots on both the outer surfaces and within the pores, thereby extending plasmonic effects and improving surface utilization, which makes mAu ideal for SERS‐based applications.^[^ ^37^ ^]^

To determine the optimum saturation point for mAu particles with 4‐MBA as the Raman reporter, a series of samples with varying concentrations of 4‐MBA were subjected to SERS analysis (Figure S5a,b, Supporting Information). The results indicate that a combination of 1 mL of mAu particles with 200 µL of 4‐MBA, at a ratio of 4:1, yields the highest signal intensity. As the plasmonic properties of nanoparticles vary with particle size, we also synthesized mAu particles with different particle sizes (40, 65, 100, and 150 nm) by adjusting the pH of micelles, in addition to the 200 nm‐sized mAu particles (Figure S6, Supporting Information). Increasing the pH with KOH enhances the reducing power of the solvent, leading to increased nucleation and a reduction in the final size of the mAu particles. Among all mAu samples, the 200 nm‐sized mAu sample exhibits well‐defined mesopores and good uniformity (Figure S7, Supporting Information) and generates the highest SERS response (Figure S8, Supporting Information). Consequently, we selected the 200 nm‐sized mAu sample for bioassay development. Furthermore, to validate the enhanced SERS signal generated from mAu, we recorded SERS spectra (Figure S9, Supporting Information) and Raman microscopic images (Figure S10, Supporting Information) of various substrates, including paper substrate, l‐cysteine, PS‐*b*‐PEO block copolymers (used for mAu preparation), mAu, 4‐MBA, and mAu SERS tag (mAu/4‐MBA/PLAP Abs). The results demonstrate that the mAu‐based SERS tag generates the highest response, while the other substrates exhibit negligible responses, confirming the formation of the SERS tag and its suitability for assay development.

### 2.3 Assay Principle

Nanoengineering has opened new possibilities in biosensing, and cancer diagnostics, particularly in situations requiring rapid and precise isolation and purification of specific biomolecules.^[^ ^38^ ^]^ To determine the specificity of PLAP in OCr, we conducted an in‐silico analysis using publicly available data from Uppsala‐Umeå Comprehensive Cancer Consortium (U‐CAN) biobank. This analysis examined the expression of PLAP across 12 different cancer types. Our analysis reveals that PLAP expression is significantly higher (*p* <0.05) in OC compared to other cancer types, including endometrial, lung, breast, cervical, chronic lymphocytic leukemia, prostate, colorectal, glioma, myeloma, diffuse large B‐cell lymphoma, and acute myeloid leukemia (Figure **2**). These findings suggest that elevated PLAP levels can serve as a potential biomarker for distinguishing OC from other malignancies, highlighting its potential clinical utility in the screening and diagnosis of OC.

In this study, we combines the magnetic properties of streptavidin‐coated beads with the unique characteristics of in‐house synthesized mAu NPs to directly isolate EVs specific to OC from patient samples and obtain enhanced SERS signals respectively. This assay avoids the need for commercial isolation kits or ultracentrifugation techniques. Leveraging their robust bio‐affinity and plasmonic properties, the mAu particles were employed to form SERS nanotag with antibodies, while the magnetic beads served as a magnetic platform for the binding of antibodies and for forming a sandwich assay with EVs and subsequently with the mAu nanotags, enabling magnetic manipulation. In this proof‐of‐concept assay, we focus on EVs, which are early indicators of cancer due to their elevated production in cancer patients. The increased release of EVs is an integral part of the intricate communication network that cancer cells utilize to facilitate their growth, progression, and interaction with the surrounding microenvironment.^[^ ^39^ ^]^

**Figure 3:**
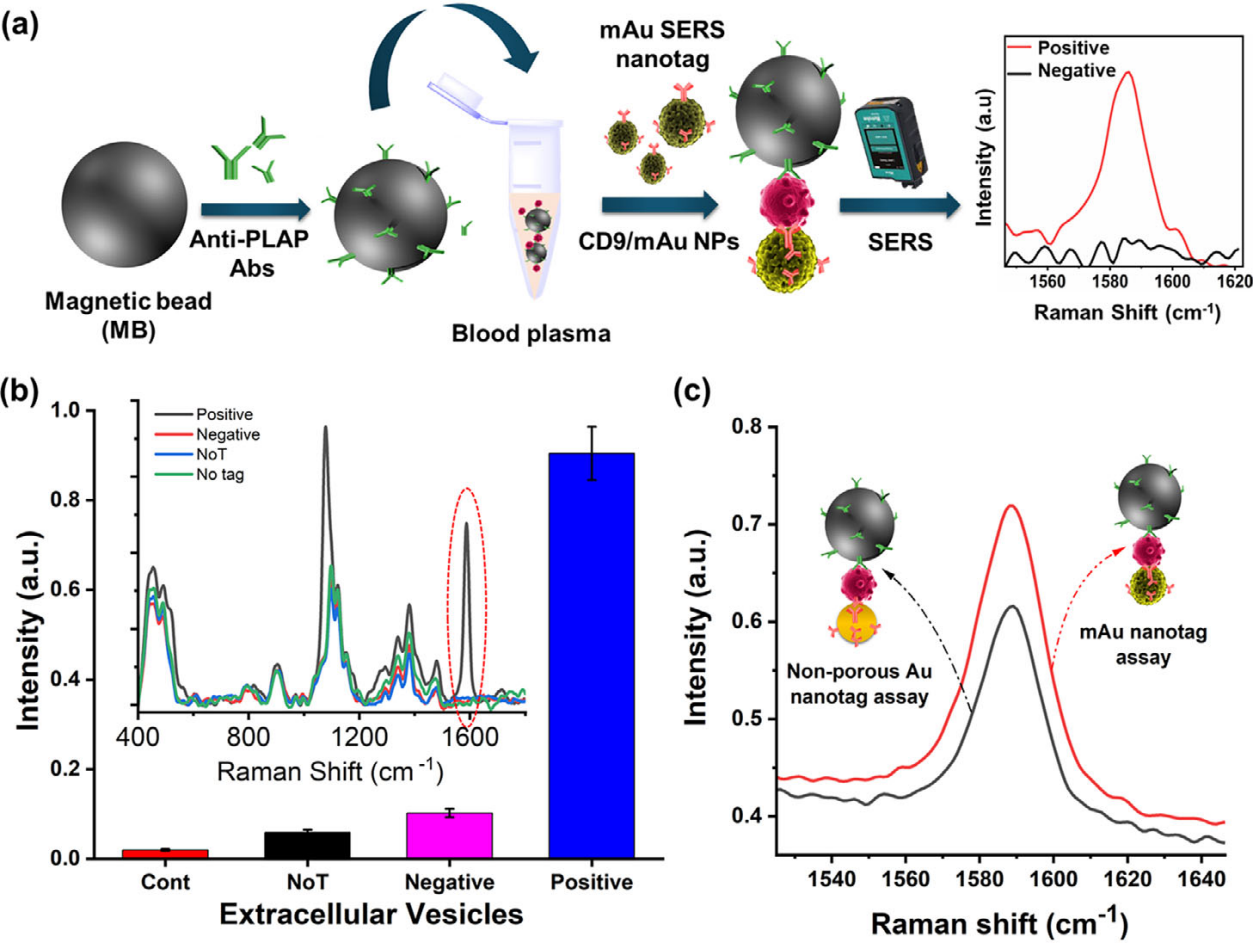
a) Schematic representation of our mAu‐based SERS assay. b) Specificity of this assay for EVs, No target (NoT), and control samples without EVs and mAu nanotags (inset; SERS spectra for each assay). c) Assay performance using both mAu‐ and non‐porous Au‐based nanotags.

A schematic representation of the mAu‐based SERS assay for EVs is depicted in **Figure** **3**a. In this assay design, streptavidin‐coated magnetic beads were initially functionalized with PLAP antibodies to isolate the PLAP^+ve^ EVs. The freshly prepared PLAP antibody‐functionalized magnetic beads were used to capture the target EVs in the samples. The use of freshly prepared PLAP‐functionalized magnetic beads was crucial to ensure that external factors did not significantly affect the assay's performance. To screen the amount of PLAP^+ve^ EVs, we employed CD‐9 functionalized with mAu nanotags, which generated distinctive SERS signals corresponding to 4‐MBA. The sandwich assay, consisting of magnetic beads attached to EVs and mAu nanotags, was quantified using a portable SERS using paper as substrate. As the amount of nanotags present in the sandwich assay directly correlates with the amount of EVs present, the intensity of the detected peaks reflects the amount of disease‐specific EVs present in the assay.

### 2.4 Assay Specificity

To assess the specificity and functionality of our assay, we conducted a series of control experiments. In the positive control trials, PLAP‐functionalized magnetic beads were initially exposed to cell culture media collected from BeWo cell (cells positive for PLAP) culture. Subsequently, mAu‐based SERS nanotags functionalized with CD9 antibodies were added to the assay. As depicted in Figure 3b, a substantial SERS intensity is detected for PLAP^+ve^ EVs derived from BeWo cells (10^7^ U mL^−1^ of total EVs isolated from cell culture media). In the negative control, we replaced PLAP with PBS. As there is no capturing antibodies with magnetic bead, no EVs is attached thereby, the control assay generates a minimum SERS response compared to the positive control. In another control experiment (Figure 3b), we used a no‐target (NoT) control by substituting PBS buffer for BeWo‐derived cell culture media. As anticipated, the NoT experiment yields a low SERS response, likely due to the physical adsorption of a negligible amount of SERS nanotag. Furthermore, in an additional control experiment without CD9, a very negligible SERS response is observed (cont.). This occurs because the absence of the CD9 antibody prevents mAu nanotags from binding to the magnetic beads in the system, resulting in their washout and a negligible SERS signal. Without the mAu nanotags, which serve as SERS signal generators, no peaks are observed. Moreover, to demonstrate the effect of porosity, we compared the performance of mAu NPs‐based assay with non‐porous Au NPs‐based assay. The assay was conducted using both mAu and non‐porous Au SERS nanotags with the same amount of total EVs (10^7^ U mL^−1^) isolated from cell culture media. The mAu NP‐based assay generates nearly twice the SERS response compared to the non‐porous Au‐based assay (Figure 3c), highlighting the advantages of the mesoporous architecture in enhancing the SERS signal. We also recorded the mapping images for both assays (Figure S11, Supporting Information), where the mAu‐based assay demonstrated more Raman hotspots, further confirming the superiority of the mAu‐based SERS assay over its non‐porous counterpart.

### 2.5 Assay Sensitivity

**Figure 4:**
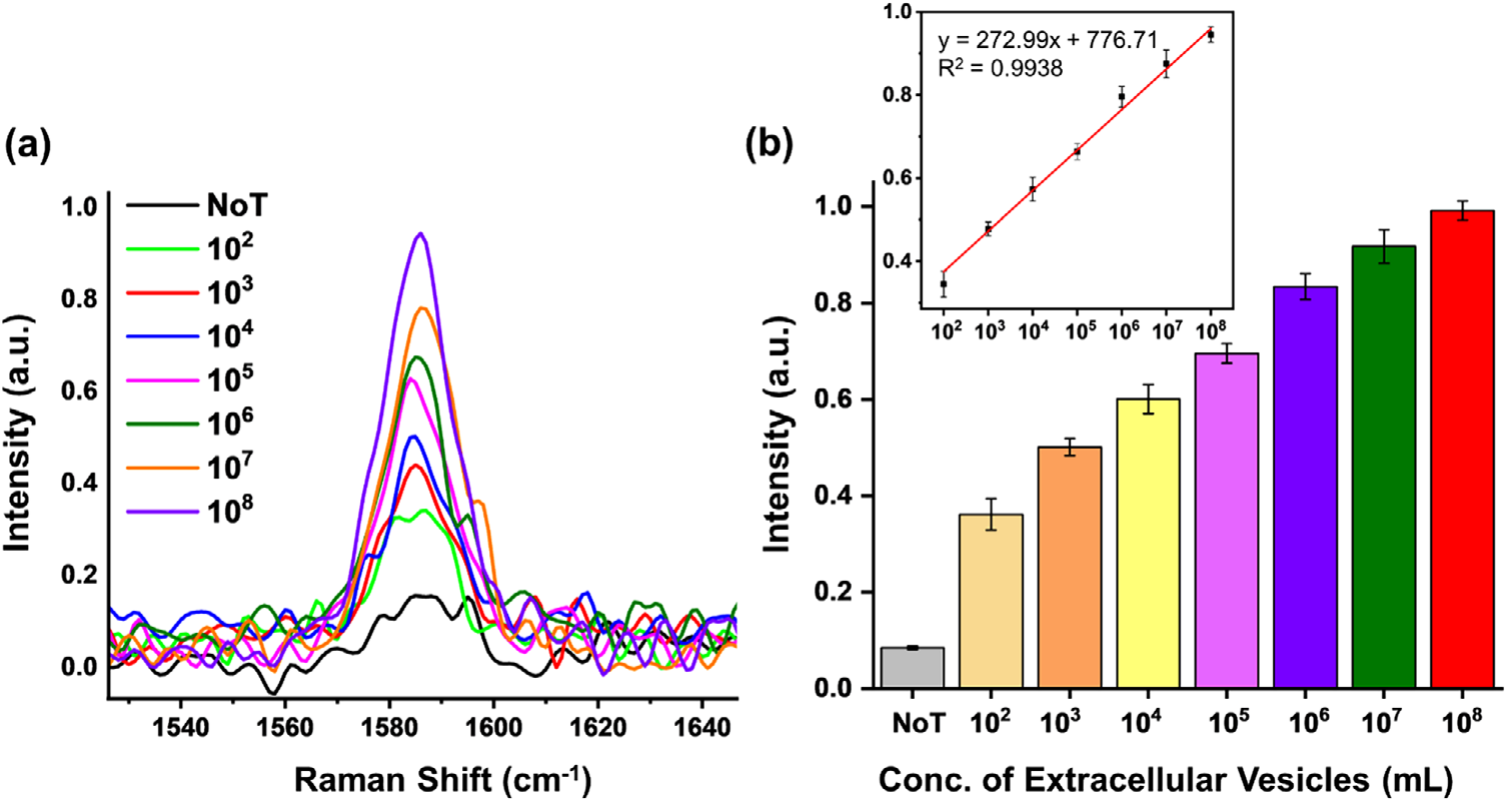
Concentration‐dependent SERS spectra for assay sensitivity. a) SERS spectra were obtained using different concentrations of EVs from 10^2^ to 10^8^ EVs mL^−1^ and b) the corresponding bar diagram. The inset in panel (b) displays the corresponding linear regression curve between the concentration of EVs and SERS intensity.

The quantity of EVs can vary depending on the stage of the cancer. In the early stage of the disease, the number of disease‐specific cells, and consequently, the quantity of EVs may be notably low. This indicates the need for a specific and sensitive approach.^[^ ^40^ ^]^ To evaluate the sensitivity of our assay, we conducted a series of experiments involving the testing of diluted positive samples obtained through serial dilution, ranging from 10^2^ to 10^8^ U mL^−1^ of total EVs isolated from cell culture media. The results show a pronounced increase in SERS intensity with increasing concentration of EVs (**Figure** **4**a). This phenomenon can be attributed to the higher quantity of target EVs, which facilitates the binding of more mAu nanotags to the immunocomplex surface, thereby creating more hotspots for SERS enhancement. As depicted in Figure 4b, a linear regression equation was fitted to the concentration of EVs versus SERS readout data, resulting in the equation *y* = 272.99*x* + 776.71, with a coefficient of determination (*R* ^2^) of 0.9938. The logarithmic concentration of EVs versus the SERS response yields a polynomial fit (Figure S12, Supporting Information). Notably, the SERS signal obtained at an EV concentration of 10^2^ is significantly higher than that of the NoT control, and the limit of detection (LOD) for our assay is estimated to be 10^2^ EVs mL^−1^. Moreover, compared to a recently reported assay utilizing noble metal nanomaterials (refer to Table S1, Supporting Information), our mAu‐based assay design demonstrates superior performance, characterized by high sensitivity and robustness.^[^ ^41^, ^42^, ^43^, ^44^, ^45^, ^46^ ^]^ Remarkably, our assay requires less than 4 h to analyze a set of samples and can be performed in an Eppendorf tube. Additionally, the incorporation of a portable SERS‐based readout enhances portability, making this assay suitable for routine clinical applications, even in resource‐limited settings.

To assess the reproducibility of our assay, we conducted three independent experiments, and the relative standard deviation (%RSD) is consistently below 6.0%. This low %RSD indicates that the isolation of EVs from plasma samples and the magnetic bead‐based SERS readout system is highly robust and reliable. This assay's ability to achieve a low LOD, while maintaining excellent reproducibility across a wide range of plasma concentrations underscores its sensitivity and specificity in detecting EVs in OC‐positive plasma samples. These results validate the effectiveness of our mAu‐based assay for the sensitive and specific detection of EVs, highlighting its potential for differential diagnosis to distingluis between benign and OC conditions.

### 2.6 Diagnostic Performance in Clinical Samples

The diagnostic performance of our assay was further validated by assessing clinical samples. A cohort of 30 plasma patient samples was analyzed, demonstrating the efficiency of our method in detecting EVs in OC patients. Plasma samples from healthy individuals (*n* = 10), benign conditions (*n* = 10), and high‐grade serous epithelial OC (*n* = 10) were processed using our developed assay. Detailed clinical characteristics of the patient samples are given in Table S2 (Supporting Information). PLAP^+ve^ EVs were isolated and purified using Anti‐PLAP antibody‐coated magnetic beads functionalized with mAu nanotags and analyzed via a SERS readout.

In this study, we used PyCaret's classification module to train fourteen different machine learning algorithms to distinguish between case and control outcomes, as previously described.^[^ ^47^ ^]^ To evaluate the performance and generalizability of the biomarker‐based models, we employed a tenfold cross‐validation strategy. The dataset was randomly partitioned into 10 equal subsets; in each iteration, nine subsets were used for training and one for testing. This process was repeated ten times, ensuring that each sample was used for both training and validation. Performance metrics, including area under the curve (AUC), precision, recall, and calibration, were averaged across all folds to generate robust and unbiased estimates of model accuracy. This approach reduces overfitting and improves the reliability of predictive performance, which is particularly important in studies with limited sample sizes.

**Figure 5:**
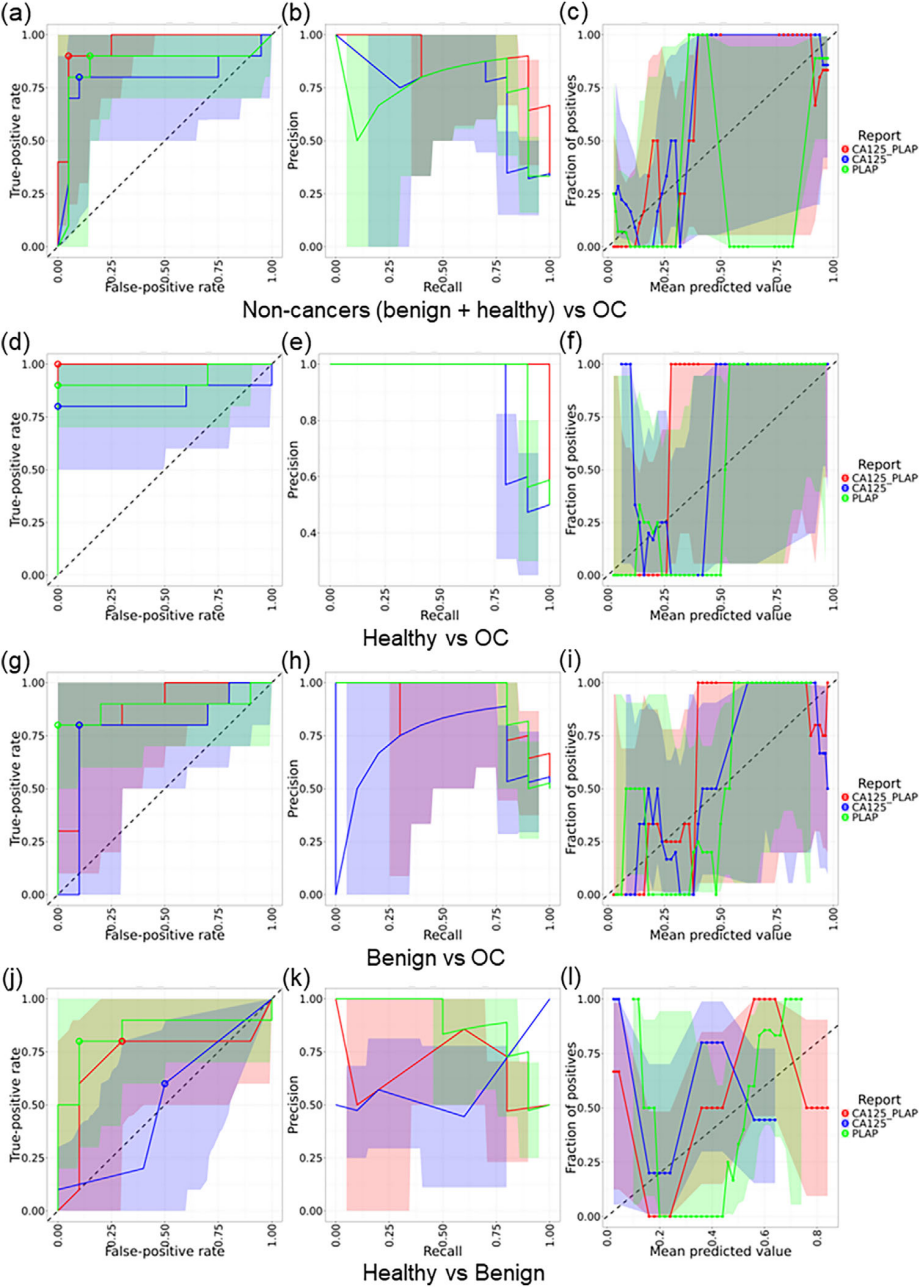
Performance evaluation of EV‐based diagnostic models using PLAP, CA125, and their combination across different pairwise comparisons. Classification performance of three biomarker‐based models: PLAP (green), CA125 (blue), and the combination CA125 + PLAP (red) to distinguish between OC, benign disease, and healthy controls. Each row represents a binary classification task, and each column shows a different performance metric: receiver operating characteristic curves (a,d,g,j), precision‐recall curves (b,e,h,k), and calibration plots (c,f,I,l).

We evaluated model performance across four classification tasks: 1) non‐cancer (benign + healthy controls) versus OC, 2) healthy controls versus OC, 3) benign versus OC, and 4) healthy versus benign. A summary of the comparative performance of PLAP, CA‐125, and their combination across these tasks is presented in **Table** **1**. Using PLAP alone, the best‐performing models achieved the following: for task 1, a specificity of 85% (95% CI: 15–100%), sensitivity of 90% (95% CI: 60–100%), and AUROC of 0.85 (**Figure** **5**a); for task 2, specificity of 100% (95% CI: 10–100%), sensitivity of 90% (95% CI: 70%–100%), and AUROC of 0.93 (Figure 5d); for task 3, specificity of 100% (95% CI: 15–100%), sensitivity of 80% (95% CI: 50%–100%), and AUROC of 0.89 (Figure 5g); and for task 4, specificity of 90% (95% CI: 15–100%), sensitivity of 80% (95% CI: 50%–100%), and AUROC of 0.84 (Figure 5j). Notably, PLAP outperformed CA‐125 in all classification tasks evaluated (Figure 5), while the combination of PLAP and CA‐125 further improved model performance in distinguishing OC from both healthy and benign controls.

**Table 1:** Comparative performance of PLAP, CA‐125, and their combination across four binary classification tasks. This table summarizes the diagnostic performance of three classifiers, PLAP alone, CA‐125 alone, and a combined PLAP + CA‐125 model, for distinguishing ovarian cancer (OC) from non‐cancer samples (benign and/or healthy controls) across four clinically relevant classification tasks. Each model was evaluated using several metrics: area under the receiver operating characteristic curve (AUROC), sensitivity and specificity with 95% confidence intervals, area under the precision‐recall curve (AUPRC), Brier score, and feature importance as determined by SHAP (SHapley Additive exPlanations) values.

Classifier	PLAP + CA‐125					
	AUROC	Sensitivity (95% CI)	Specificity (95% CI)	AUPRC	Brier score	SHAP
Benign + Healthy vs OC	0.95	90% (20–100%)	95% (60–100%)	0.90	0.10	PLAP, CA‐125
Healthy vs OC	1.00	100% (100–100%)	100% (100–100%)	1.00	0.06	PLAP, CA‐125
Benign vs OC	0.87	80% (10–100%)	90% (40–100%)	0.86	0.14	CA‐125, PLAP
Healthy vs Benign	0.72	80% (40–100%)	70% (0–100%)	0.67	0.21	PLAP, CA‐125

Classifier	CA‐125					
	AUROC	Sensitivity (95% CI)	Specificity (95% CI)	AUPRC	Brier score	SHAP
Benign + Healthy vs OC	0.79	80% (13–100%)	90% (5–100%)	0.74	0.13	CA‐125
Healthy vs OC	0.84	80% (50–100%)	100% (15–100%)	0.91	0.13	CA‐125
Benign vs OC	0.77	80% (0–100%)	90% (10–100%)	0.68	0.17	CA‐125
Healthy vs Benign	0.50	60% (0–88%)	50% (17–80%)	0.63	0.28	CA‐125

Classifier	PLAP					
	AUROC	Sensitivity (95% CI)	Specificity (95% CI)	AUPRC	Brier score	SHAP
Benign + Healthy vs OC	0.85	90% (60–100%)	85% (15–100%)	0.73	0.11	PLAP
Healthy vs OC	0.93	90% (70–100%)	100% (10–100%)	0.96	0.09	PLAP
Benign vs OC	0.89	80% (50–100%)	100% (15–100%)	0.93	0.15	PLAP
Healthy vs Benign	0.84	80% (30–100%)	90% (15–100%)	0.88	0.21	PLAP

To further assess the clinical reliability of the models, we examined recall and calibration curves, which are critical for evaluating sensitivity across thresholds and the accuracy of predicted probabilities. The combined PLAP and CA‐125 model consistently achieved higher and more stable recall, along with well‐calibrated predictions, particularly in tasks differentiating OC from healthy and non‐cancer controls (Figure 5b,c). PLAP alone showed comparable calibration and recall in the OC versus healthy comparison, whereas CA‐125 alone yielded more variable recall and poor calibration, particularly in the benign versus OC and healthy versus benign comparisons (Figure 5e,f). Across all models, performance was lowest in distinguishing benign from healthy samples, as reflected by fluctuating recall values and miscalibrated predictions (Figure 5a,l). These findings demonstrated that PLAP is a more robust biomarker than CA‐125 for detecting OC in the cohort of patients used in this study, and that integrating PLAP with CA‐125 improves diagnostic accuracy and reliability, supporting its potential utility in developing clinically actionable, non‐invasive diagnostic tools for detection of OC.

In summary, our analysis demonstrates clear differences in biomarker profiles among healthy controls, patients with benign conditions, and those with OC, with the most pronounced separation observed between OC and non‐cancer groups. A moderate but consistent distinction was also noted between healthy and benign samples. These findings highlight the strong discriminatory power of our assay, particularly in differentiating ovarian cancer from non‐malignant conditions, underscoring its diagnostic potential. While the clinical utility of the assay was validated in a small cohort through the sensitive detection of PLAP‐positive EVs, our results support its scalability and suggest that an optimized version could be applied to larger patient cohorts. Importantly, this platform has the flexibility to extend beyond EV detection in OC and could be adapted as a broadly applicable biosensing tool for other cancers and chronic diseases.

## 3 Conclusion

In this study, we have successfully developed a nanostructured SERS‐based assay using mAu NPs to detect and quantify PLAP^+ve^ EVs for OC diagnosis. These mAu particles significantly enhance antibody loading and SERS probe efficiency, improving the platform's sensitivity. The mesoporous architecture enhances the detection sensitivity by generating more SERS hotspots, leading to stronger signals. Through proof‐of‐concept analysis, the assay demonstrates its practicality in clinical settings by effectively isolating EVs from OC patients, benign cases, and healthy controls. With a low LOD (10^2^ EVs mL^−1^), our assay is proven to be highly sensitive, showing significantly higher EV levels in OC patients compared to benign and healthy controls. Notably, the quantification of PLAP^+ve^ EVs reveals distinct differences between OC patients and non‐cancer controls, suggesting the specificity of these EVs for OC and oncogenic transformation. The assay achieves a sensitivity of 82% and a specificity of 98%, accurately identifying ovarian cancer patients through cross‐validation, highlighting its potential for differential diagnosis for OC. This platform offers a minimally invasive, efficient, and reliable tool for detecting OC, thereby enabling timely interventions and improving patient outcomes. However, while PLAP^+ve^ EVs hold promise, it is crucial to recognize that PLAP expression is not exclusive to OC. Further research is required to fully establish its diagnostic value in this context. This study underscores the clinical relevance and translational potential of EV‐based biomarkers for OC diagnosis and management. With additional clinical validation in a larger patient cohort, this assay have the potential to be integrated into clinical practice, providing a valuable tool for detection, disease monitoring, and personalized treatment strategies due to its sensitivity and specificity.

## 4 Experimental Section

### Synthesis of mAu

The mAu particles were prepared according to the previous report^.[^ ^35^ ^]^ In a typical synthesis, 1.25 mg of polystyrene‐*b*‐poly(ethylene oxide) (PS_18000_‐*b*‐PEO_7500_) was completely dissolved in 0.25 mL of THF at 40 °C. Next, 125 µL of ethanol, 57 µL of 750 mm KOH, and 127 µL of an aqueous 10mm l‐cysteine solution were sequentially added to the block copolymer solution and stirred gently for 20 min in an ice bath. After that, a cold aqueous solution of 80 mm HAuCl_4_ (188 µL) and 250 µL of 100 mm AA were consecutively added to the above mixture solution and stirred for 2 min. In this timeframe, the color of the solution changed from dark transparent yellow to colorless, and then from red to blue indicating the formation of the final product. The reaction mixture was stirred for another 1 h in the ice bath. The samples were collected by centrifugation at 14 000 rpm for 15 min, washed using THF/ethanol (1:1). This washing and centrifugation process was repeated 8 times to ensure complete removal of the polymeric micelle templates.

### Preparation of Non‐Porous Au

Initially, Au NPs with a mean diameter of 30 nm were synthesized to serve as seeds for the generation of larger NPs. Under gentle stirring, a solution containing polyvinylpyrrolidone (PVP) (280 mm) and HAuCl_4_ (5 mm) was added to a flask. Then, 0.5 mL of 30 mm AA was rapidly added to the mixture. The solution changed from colorless to red, signifying the formation of small Au nanoparticles (“seed solution”). Next, in a separate 2 mL vial, 227 µL of 80 mm HAuCl_4_, 39.6 µL of water, and 176 µL of 40 mm sodium citrate (Na_3_Ctr) were gently mixed. Following this 150 µL of the seed solution was added, and the mixture was stirred at an ambient temperature for 1 h. The resulting product was washed with water and centrifuged at 14 000 rpm for 5 min.

### Preparation of SERS Tags

To create SERS nanotags, a thiol monolayer of dithiobis (succinimidyl propionate) (DSP) and Raman reporters were added, followed by conjugation with secondary detection antibodies, as per previously established protocol.^[^ ^48^ ^]^ Briefly, 2 µL of 1.0 mm DSP in dimethyl sulfoxide (DMSO) and 100 µL of 200 mm 4‐MBA (4‐ mercaptobenzoic acid) in ethanol were incubated with 1 mL of mAu particles for ≈12 h under magnetic stirring. In the presence of DSP, mAu particles were combined with the Raman reporters to create SERS nanotags. The amine groups of the antibody protein bonded to the succinimidyl ester of DSP, while the Raman reporters were attached to the surface of mAu particles via chemisorption of the aromatic thiol. Following this, the supernatants were removed by centrifuging the colloid for 15 min at 6500 rpm. The mAu particles were then resuspended in 200 µL of PBS buffer. To conjugate the specific detection antibody (anti‐CD9), 200 µL of the antibody solution was added to the colloid, which was then incubated for 1 h at ambient temperature. Before being resuspended in 200 µL of 0.1% bovine serum albumin (BSA) for 30 min at room temperature, the colloid was centrifuged again for 15 min at 6500 rpm at 4 °C to remove any unconjugated antibodies. BSA was added to stabilize the SERS nanotags, preventing aggregation, and minimizing non‐specific protein binding on the SERS surface.

### mAu‐Based SERS Assay for EV Detection

Magnetic beads (5 µL) were first functionalized with PLAP antibody (5µL, 1 µM) to isolate PLAP‐specific EVs present in plasma (or cell culture media) samples (10 µL) extracted from OC patients. After the isolating and purifying the EVs, as prepared mAu nanotags (5 µL) were used to form a sandwich assay with the prepared mAu nanotags. A portable SERS (Metrohm MiraCal DS Raman spectrophotometer) readout was then used to quantify the sandwich assay, thereby amount of PLAP^+ve^ EVs present in the sample. 0.1% bovine serum albumin (BSA) was used as a blocking agent to hinder the adsorption of non‐specific EVs to the anti‐PLAP antibodies/magnetic beads. Each steps was followed by three washing steps using a magnet block. The final immunocomplex was resuspended in 10 µL of 1 mM PBS and a 5 µL aliquot of the solution was placed upon the substrate for spectral analysis.

### Expression of PLAP In Plasma

The normalized protein expression (NPX) dataset consists of plasma profiles of 1464 proteins from over 1300 patients with various cancers, derived from the Uppsala‐Umeå Comprehensive Cancer Consortium (U‐CAN) biobank.^[^ ^49^ ^]^ The values for Alkaline phosphatase, Placental (UniProt accession: P05187) were extracted and plotted across 12 different cancer types (*n* = 12).

### Patient‐Derived Specimens

Blood samples were collected from women prior to surgery for suspected ovarian adenocarcinoma using an Oregon Health & Science University (OHSU) Institutional Review Board approved protocol (OHSU #0921). Each participant provided informed consent for a blood draw (10 mL in sodium‐citrate tubes). This study also received approval by the Research Ethics Committee of The University of Queensland (2020/HE001852). Platelet poor plasma was prepared by centrifugation at 2500 × g for 10 min, and 0.5 mL aliquots were stored at −80 °C. These banked, coded plasma samples were provided by OHSU for experimental analysis, linked to a coded database without patient health identifiers, as extracted from electronic medical records. Coded subject data included patient age, pathological diagnosis (e.g., benign cystadenoma, borderline tumor, primary ovarian carcinoma including differentiation and grade, or metastatic carcinoma), which was confirmed by a board‐certified gynecologic pathologist (Dr. Morgan, OHSU),^[^ ^50^ ^]^ and clinical stage. Plasma samples from patients diagnosed with high‐grade serous epithelial OC were used in this study.

### Model Construction and Evaluation

To develop predictive models for classifying case and control outcomes, PyCaret's classification module (version 3.3.2), a low‐code Python‐based machine learning library that streamlines the end‐to‐end model development workflow was employed as previously described.^[^ ^47^ ^]^ All analyses were conducted using Python version 3.11.0. A total of fourteen supervised machine learning algorithms were evaluated: Random Forest (RF), Support Vector Machine with Radial Basis Function Kernel (RBFSVM), Extra Trees Classifier (ET), Gaussian Process Classifier (GPC), Decision Tree Classifier (DT), Multi‐layer Perceptron (MLP), Light Gradient Boosting Machine (LIGHTGBM), K‐Nearest Neighbors (KNN), Gradient Boosting Classifier (GBC), AdaBoost Classifier (ADA), Logistic Regression (LR), Linear Discriminant Analysis (LDA), Quadratic Discriminant Analysis (QDA), and Naive Bayes (NB). To ensure robust model evaluation and to minimize overfitting, a tenfold cross‐validation strategy was applied using the entire dataset. In this approach, the dataset was randomly partitioned into 10 equal subsets; in each iteration, nine subsets were used for training and one for validation. This process was repeated 10 times, ensuring that each sample was used for validation exactly once. Performance metrics—including accuracy, sensitivity, specificity, positive predictive value (PPV), negative predictive value (NPV), area under the receiver operating characteristic curve (AUROC), area under the precision‐recall curve (AUPRC), and Brier score, were averaged across all folds to obtain robust performance estimates for each algorithm. Model selection was primarily based on cross‐validated accuracy. For the best‐performing model, this study further computed feature importance to identify the most influential biomarkers contributing to classification performance. This analysis provides valuable insights into which variables most effectively differentiate between control and case samples, supporting the biological relevance and diagnostic potential of the selected features.

## Conflict of Interest

The authors declare no conflict of interest.

## Supporting information



Supporting Information

## Data Availability

The data that support the findings of this study are available in the supplementary material of this article.
