# Supplementary material for: Plasmonic Mesoporous Gold‐Based SERS Biosensor for Ovarian Cancer‐Derived Extracellular Vesicles
Source: Small. 2025 Jul 9;21(34):2401817. doi: 10.1002/smll.202401817 (PMC12393012; doi:10.1002/smll.202401817)
Supplement: Supplementary file 1 — Supporting Information [file SMLL-21-2401817-s001.docx]

**Supporting Information**

***For***

**Plasmonic Mesoporous Gold-based SERS biosensor for Ovarian Cancer-Derived Extracellular Vesicles**

Javeria Bashir, Mostafa Kamal Masud*, Asep Sugih Nugraha, Chia-Hung Liu, Arya Vasanth, Aditya Ashok, S M Azad Hossain, Emtiaz Ahmed, Tanja Pejovic, Terry Morgan, John Hooper, Andrew Lai, Dominic Guanzon, Yusuf Valentino Kaneti, Md Shahriar A. Hossain*, Carlos Salomon Gallo*, Yusuke Yamauchi*

# **Supporting Experimental Section**

**Cell culture and isolation of** **Extracellular Vesicles**

PLAP-positive BeWo choriocarcinoma cell lines were purchased from ATCC (USA). The cells were cultured in 75-cm^2^ flasks (Life Technologies, Australia) at 37 °C and 5% CO_2_ in high-glucose DMEM containing GlutaMAX^TM^ (Life Technologies, Australia), supplemented with 10% exosome-depleted fetal bovine serum (Life Technologies, Australia) and 1% penicillin/streptomycin (Life Technologies, Australia). The cell culture medium, containing exosomes, was harvested after 72 h of incubation.

**Enrichment of Extracellular Vesicles (EVs)**

The culture of BeWo cells and exosome isolation procedures were conducted in an ISO17025 accredited (National Association of Testing Authorities, Australia) research facility. All data were recorded in compliance with 21 Code of Federal Regulation (CFR) part 11 using an electronic laboratory notebook (Irisnote, Redwood City, CA, USA). The BeWo human choriocarcinoma cell line was purchased from the European Collection of Cell Cultures (Porton Down, Salisbury, United Kingdom). Cells were maintained in phenol red-free RPMI 1640 medium, supplemented with 10% heat-inactivated fetal bovine serum, 1% non-essential amino acids, 1 mM sodium pyruvate and 100 U/mL penicillin, and 100 mg/mL streptomycin. Cultures were incubated at 37 °C in a humidified atmosphere with 5% CO_2_. Cells were sub-cultured with dissociation media, TrypLE^TM^ Express (Life Technologies, USA) and cell viability was determined using Trypan Blue exclusion and a Countess^®^ Automated cell counter (Life Technologies, USA). EVs were isolated from BeWo cells-conditioned medium as previously described,^S1-S4^ with slight modifications. Briefly, samples were centrifuged sequentially at 800 x *g* for 10 min, 2,000 x *g* for 10 min, and 12,000 x *g* for 10 min to remove whole cells and debris from the AT-conditioned media. The resulting supernatant fluid was passed through a 0.22 μm sterile filter (Steritop™, Millipore, Billerica, MA, USA) and then centrifuged at 100,000 x *g* for 120 min (Thermo Fisher Scientific Inc., Asheville, NC, USA, Sorvall, SureSpinTM 630/36, Tube angle: 900). The resulting 100,000 g pellet was resuspended in 300 μL of PBS for size exclusion chromatography (SEC) using in-house prepared columns. Briefly, Pierce™ Disposable Columns, 10 mL (Thermo Scientific) were packed with 10 mL of Sepharose® CL-2B (Sigma) beads and left overnight at 4 °C to form a packed bed. The packed bed was equilibrated with ice-cold PBS and topped with a column filter. The 300 µL of clarified plasma was applied to the top of the column, followed by PBS elusion. Then, 500 μL of 12 fractions were collected, and particle concentration were determined using both nanopore analysis and nanoparticle (NPs) tracking analysis (NAT, NanoSight). High-particle fractions were pooled and stored at −80 °C until exosome analysis.

# **Supplementary Figures**


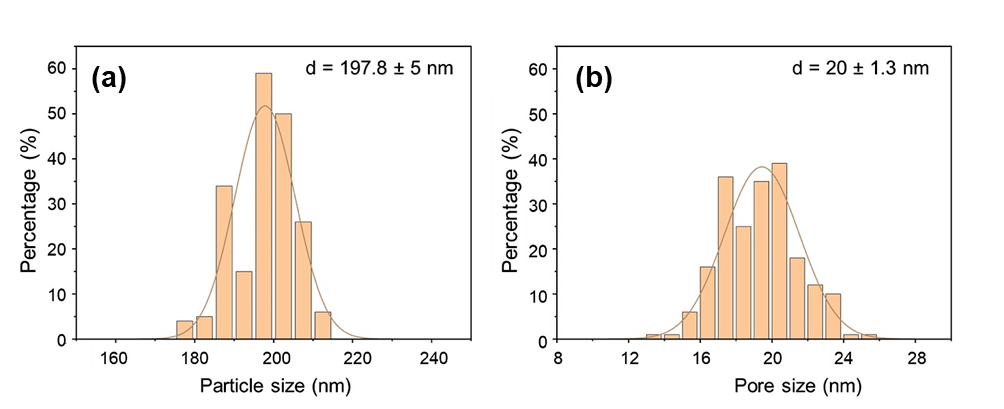


**Figure S1.** (a) Particle size and (b) pore size distribution histograms of mAu NPs.

**
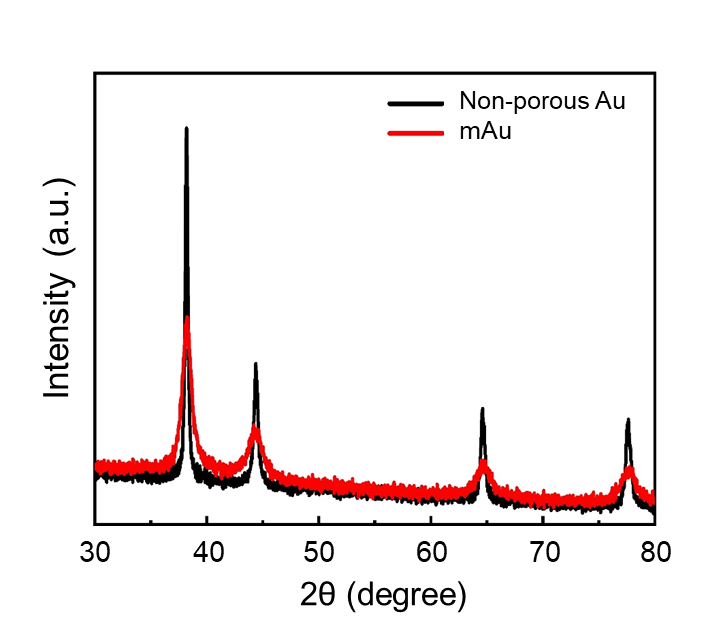
**

**Figure S2.** Wide-angle X-ray diffraction (XRD) patterns of mAu NPs and non-porous Au NPs.

***Notes***: The XRD shows the (111), (200), (220) and (311) planes of Au. The reduction in XRD peak intensity and peak broadening for mAu NPs occurs due to the mesostructuring process.


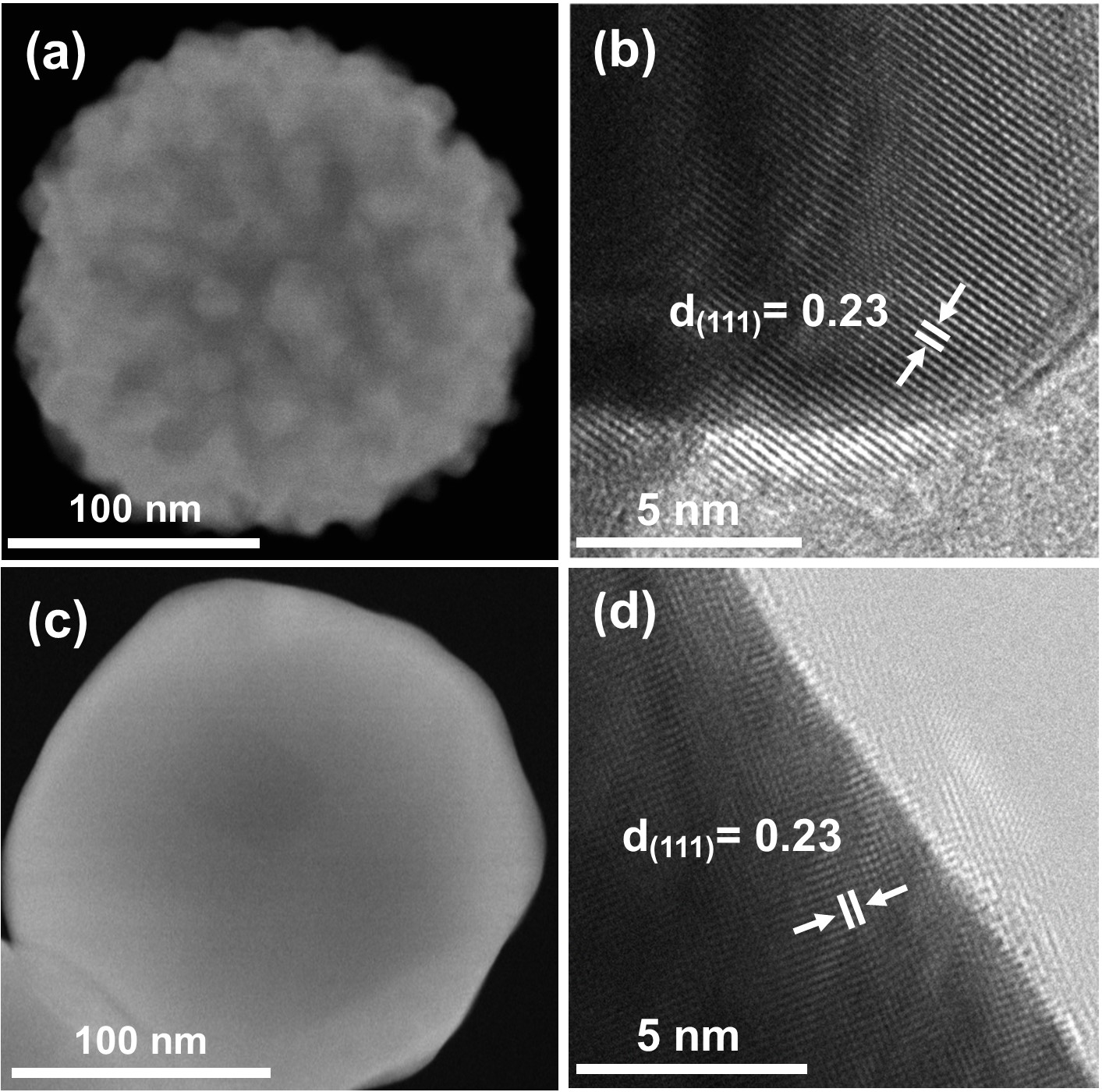


**Figure S3.** TEM and high-resolution TEM (HRTEM) images of (a, b) mAu NPs and (c, d) non-porous Au NPs.

**
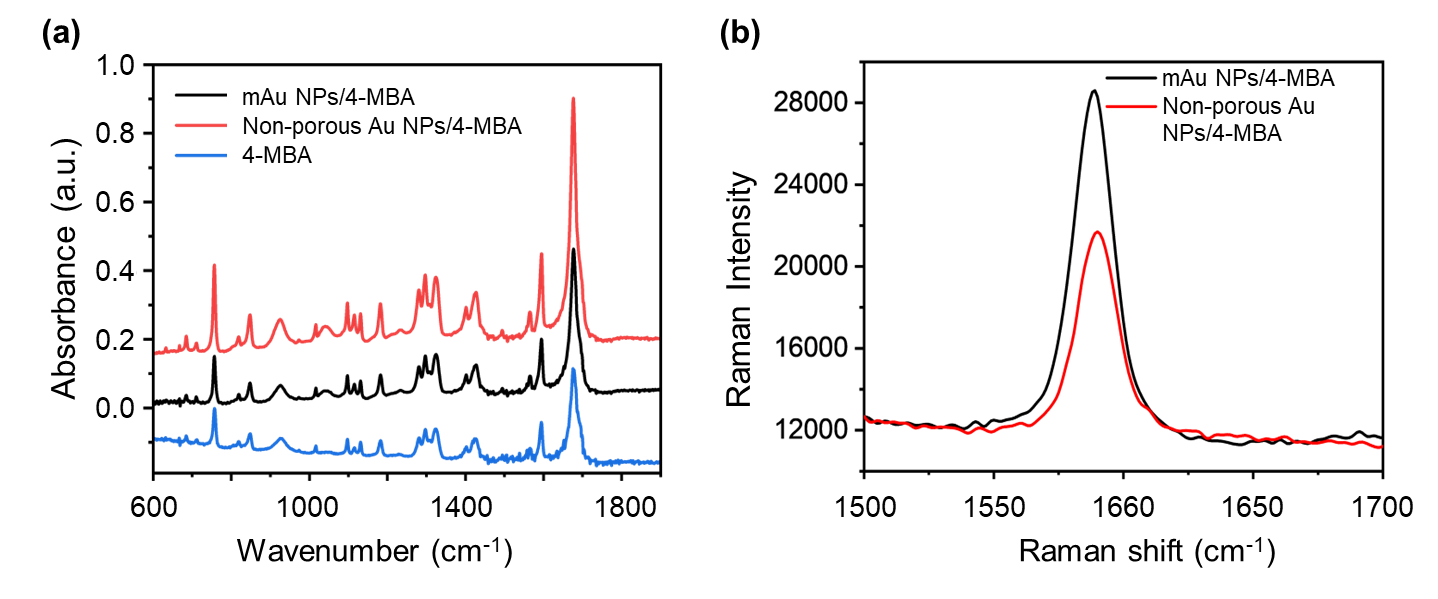
**

**Figure S4.** (a) IR spectra for 4-mercaptobenzoic acid (4-MBA), mAu NPs, and non-porous Au NPs labeled with 4-MBA. (b) Raman spectra of mAu NPs and non-porous Au NPs labeled with 4-MBA, measured at a wavelength of 785 nm.

**
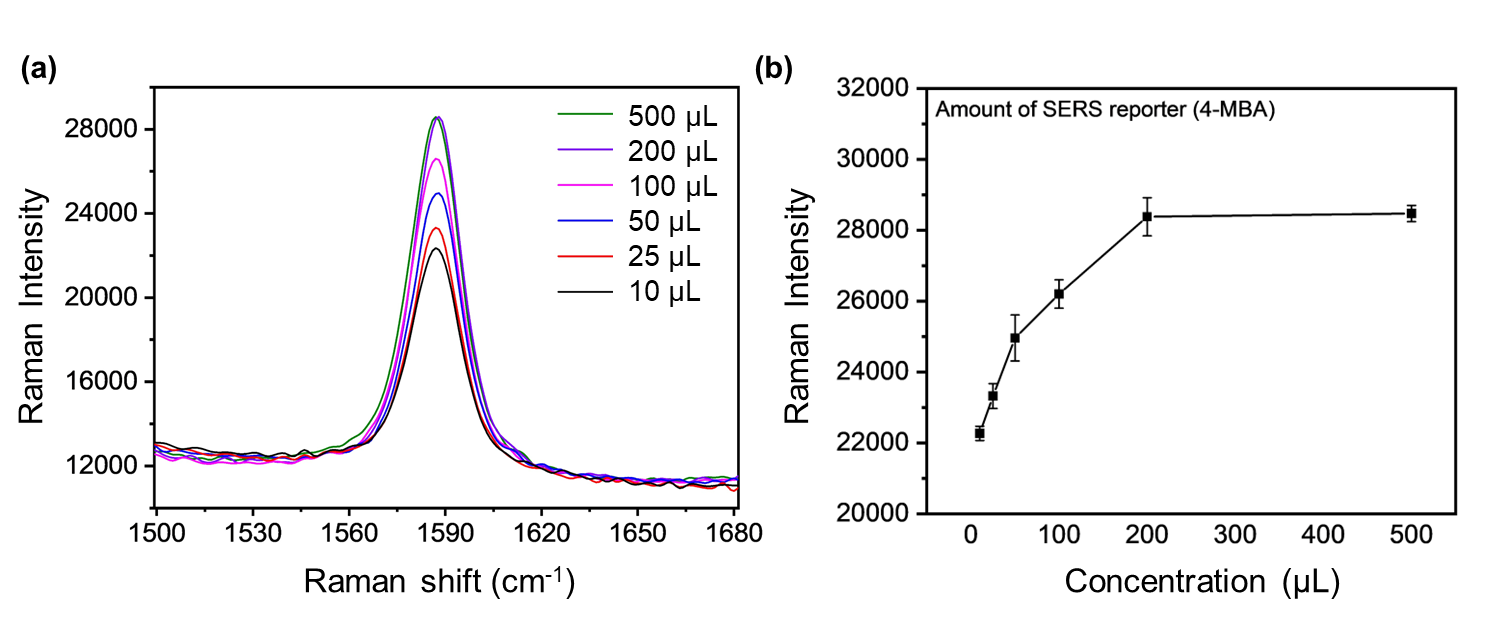
**

**Figure S5.** (a, b) Optimization of 4-MBA in mAu performed at various concentrations ranging from 10 µL to 500 µL. The SERS signals consistently increase up to 200 µL, before stabilizing.

***Notes***: This observation suggests that increasing the amount of 4-MBA beyond 200 µL does not result in further enhancements, indicating that at this concentration, the mAu NPs are fully saturated with 4-MBA molecules.


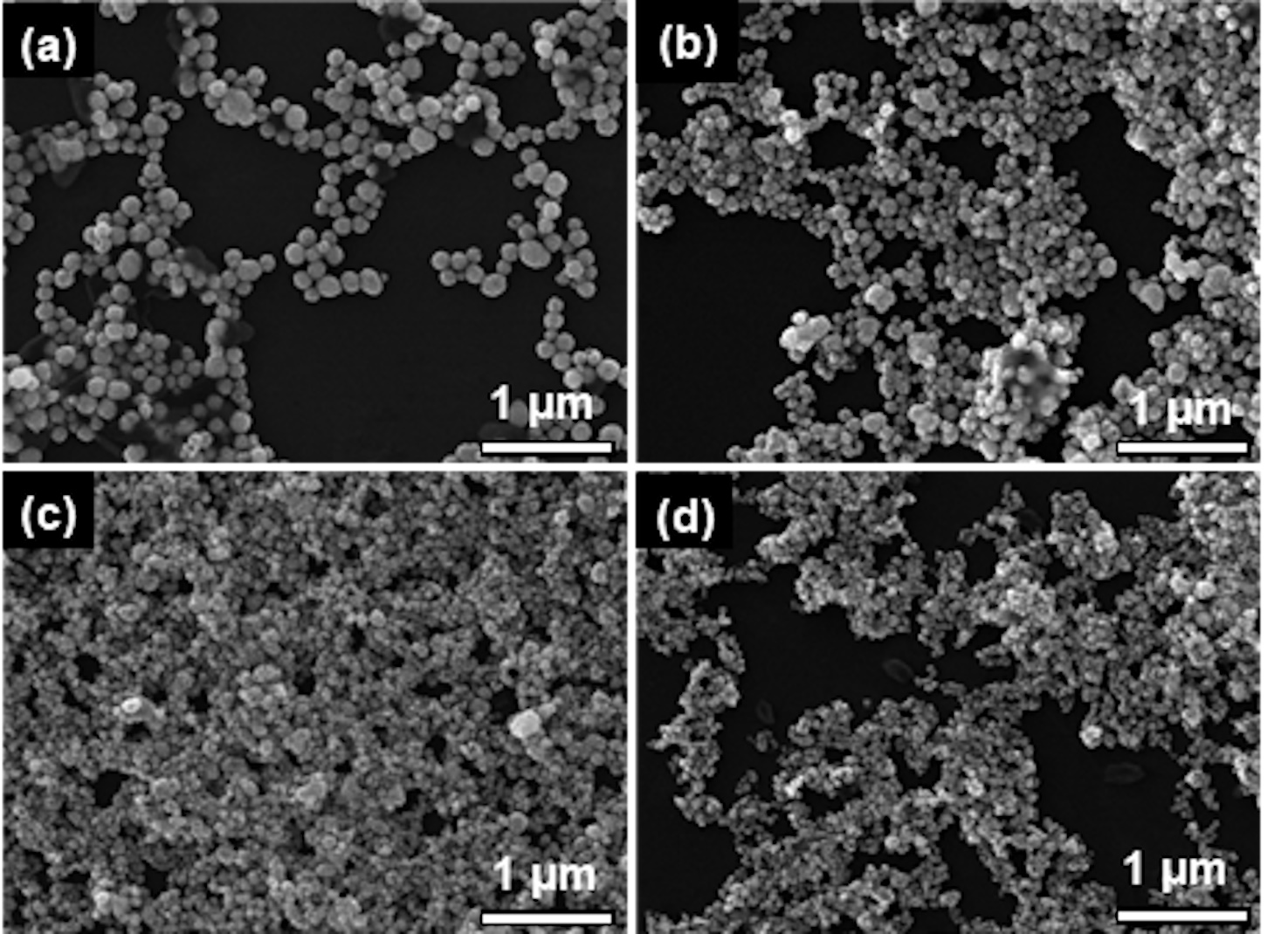


**Figure S6.** Low-magnification SEM images of mAu NPs with different particle sizes: (a) 150 nm, (b) 100 nm, (c) 65 nm, and (d) 40 nm


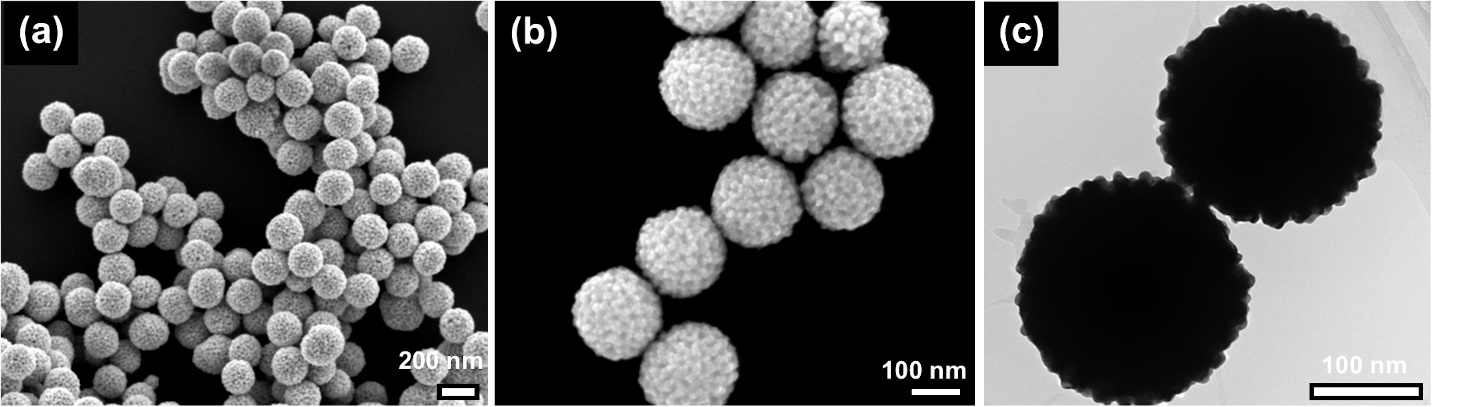


**Figure S7.** (a) Low- and (b) high-magnification SEM, and (c) TEM images of 200 nm-sized mAu NPs.


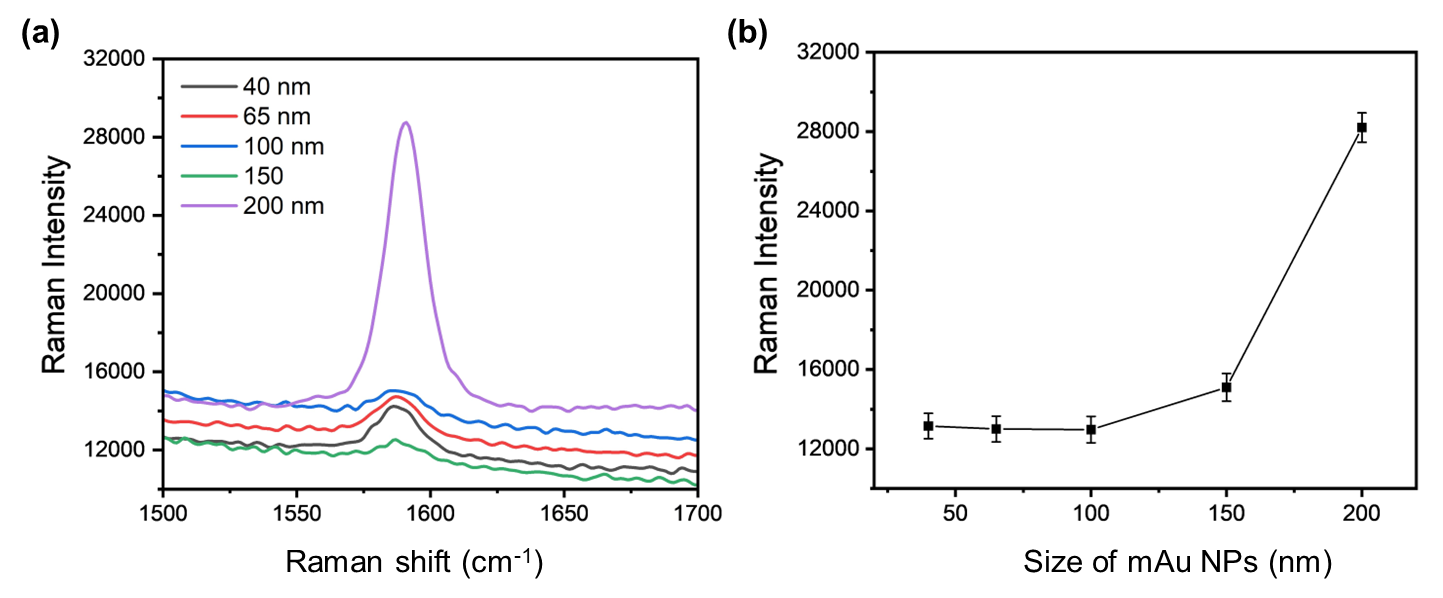


**Figure S8.** (a) Raman intensity plotted as a function of particle size for mAu NPs and (b) the corresponding line plot showing the trend in Raman intensity variation with particle size.


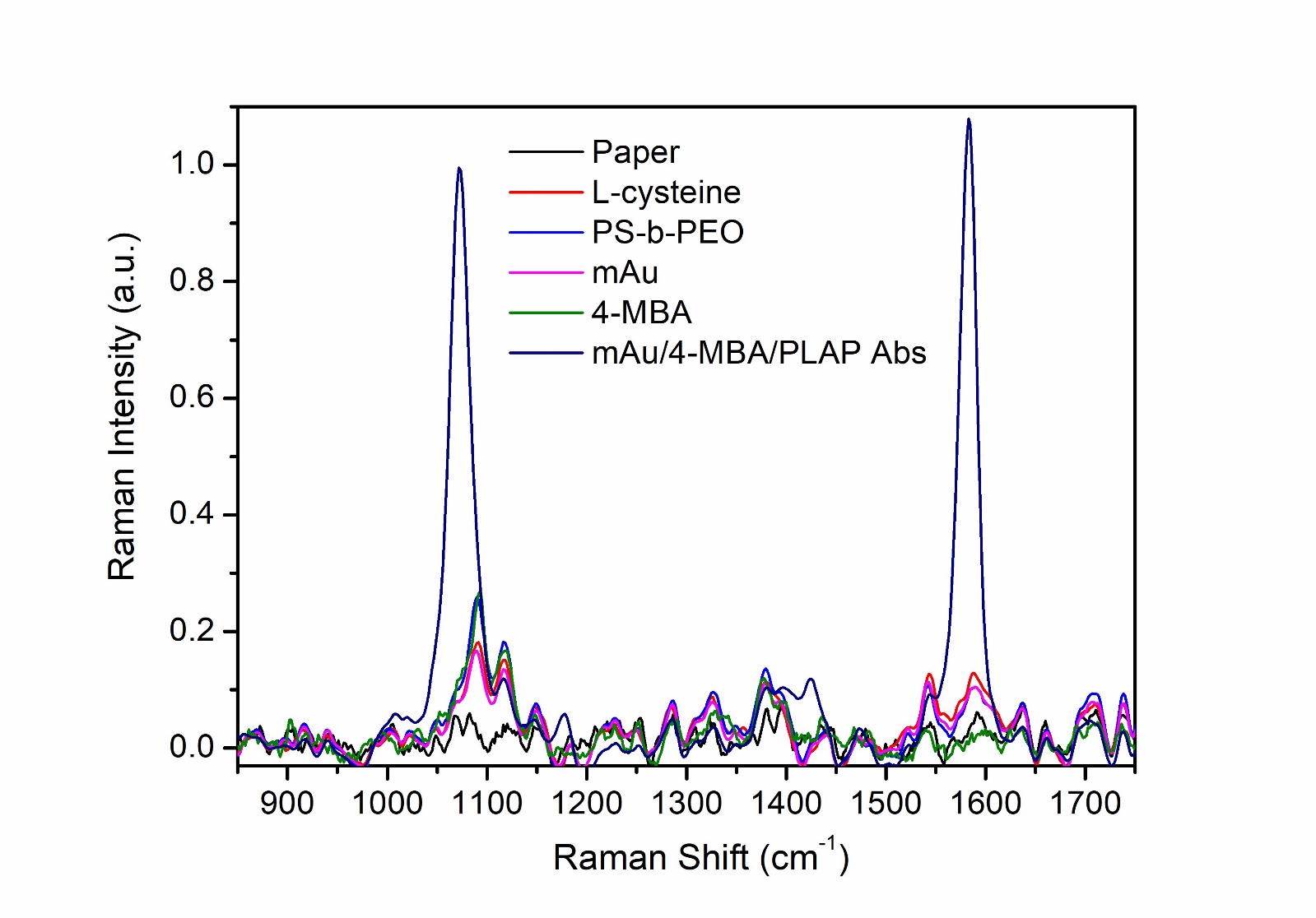


**Figure S9.** Typical SERS spectra of various control experiments. The characteristic SERS spectra of filter paper, L-cysteine, polymer (PS-*b*-PEO), mAu NPs, Raman reporter (4-MBA), and functionalized SERS nanotags (mAu/4-MBA/PLAP Abs). Each sample was separately absorbed onto filter paper and scanned independently using the MIRA SERS spectrometer. The spectra provide a comparison of the distinct Raman signals generated by each component.


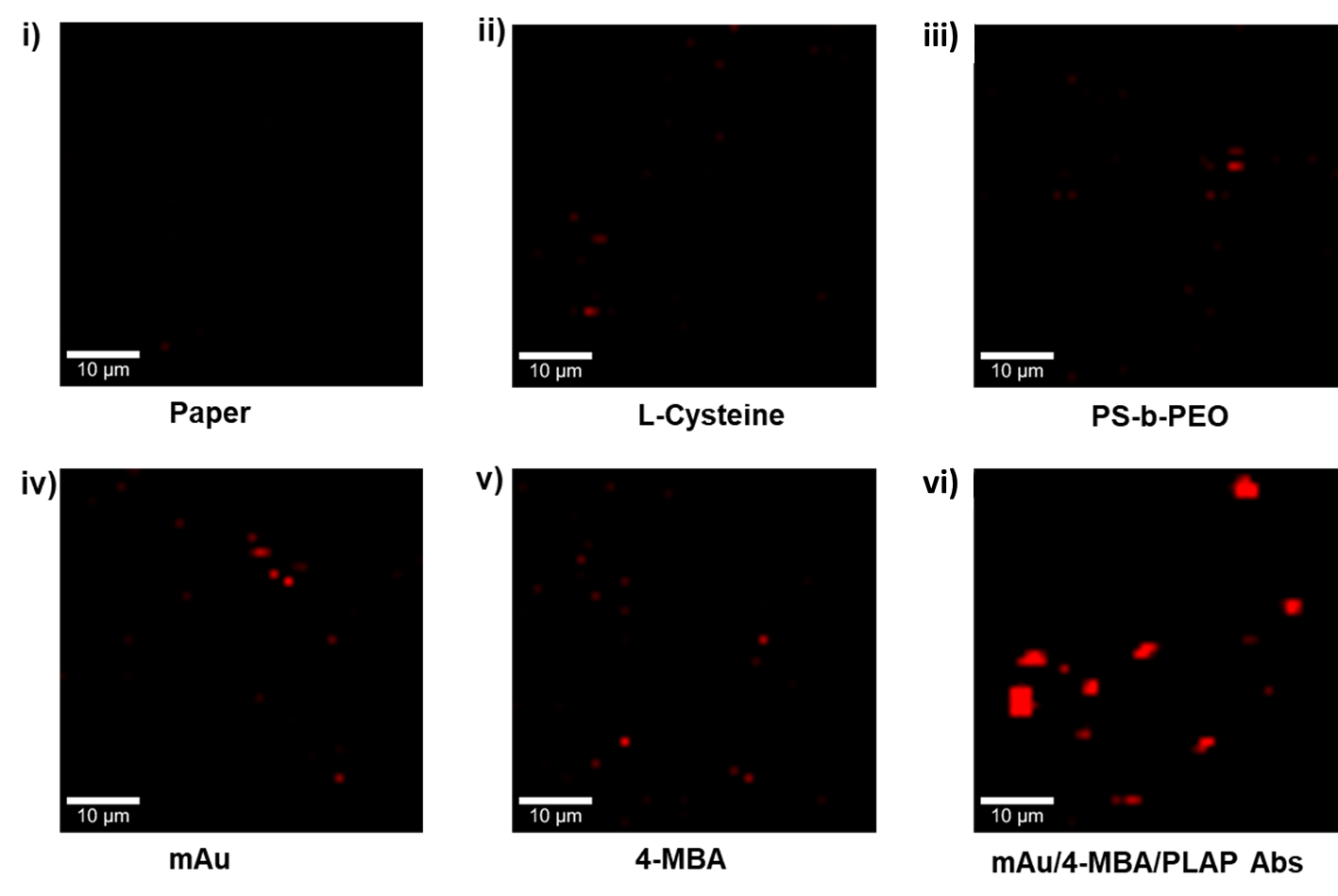


**Figure S10.** SERS mapping images of i) filter paper, ii) L-cysteine, iii) polymer (PS-*b*-PEO), iv) mAu NPs, v) Raman reporter (4-MBA), and vi) functionalized SERS nanotags (mAu/4-MBA/PLAP antibody). These images were taken using Witec alpha 300 R microscope with a 20X objective).

.
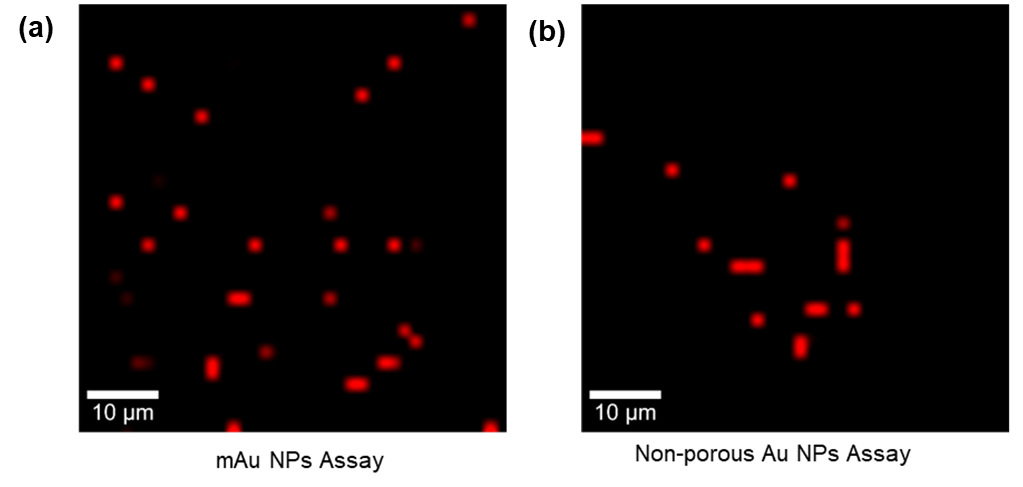


**Figure S11.** SERS mapping images for (a) mAu-NPs and (b) non-porous Au NPs based assay. These images were taken using Witec alpha 300 R microscope with a 20X objective).


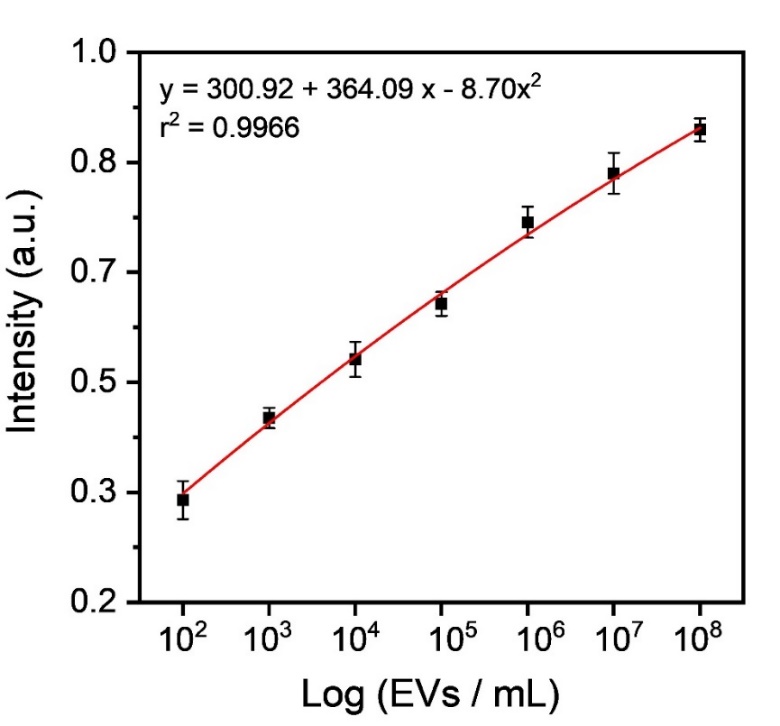


**Figure S12.** Polynomial fit of the logarithm of EV concentration (ranging from 10^2^ to 10^8^ EVs/mL) *versus* the SERS response.

**Table S1.** Nanomaterial-based detection of exosomes and exosomal biomarkers.

| **Nanostructures** | **Function** | **Assay Principle** | **Linear range** | **LOD** | **Ref.** |
| --- | --- | --- | --- | --- | --- |
| C-Fe_2_O_4_ | Dispersible nanocarriers and nanozymes | C-IONPs for the direct capture and electrochemical detection of exosomes derived from ovarian cancer cell. | 6.25×10^5^ - 1.0×10^7^ exos/mL | 1.25×10^6^  exo/mL | S5 |
| AuNS@Au-MBs | Isolation and quantification of exosomes | Exosomes, targeted through antibodies on modified magnetic beads, form sandwich-type complexes with SERS nanoprobes quantitative detection through Raman signals. | 40 - 4 × 10^7^ particles/μL | 27 particles/µL | S6 |
| Au@Ag nanobipyramids and Au nanorods | Plasmonic signal generators | Capturing exosomes with Au@Ag NPs, in a sandwich immunoassay by secondary labeling with Au NRs. | 1.2×10^3^ particles/μL -6.3×10^3^ particles/μL | - | S7 |
| MB@SiO_2_@Au and AuNP@aptamer | Magnetic isolation and SERS signals generators | Magnetic capturing substrates (MB@SiO_2_@Au@aptamer) functionalized with aptamer, can specifically capture enabling the formation of a sandwich-type apta-immunocomplex. |  | 32-203 exosomes/µl | S8 |
| Au film and AuNPs | Signal amplification | AuNP, as well as utilizing a dual AuNP amplified SPR aptasensor for exosome detection. |  | 5 × 10^3^ exosomes/mL | S9 |
| Fe_3_O_4_ NPs | Separation and quantification | PSMA (prostate specific membrane antigen) aptamer was applied to capture PSMA-positive exosomes and single strand DNAs with aptamers were divided into two parts to decrease the hybridization energy with aptamers, and further increase the competition capacity of exosomes. | 10^3^ to 10^8^ /μL | 100 particles/μL | S10 |
| Au NPs | SERS signal generator | A CD63 aptamer, blocked by a DNA probe, is utilized for capture of CD63-bearing exosomes and linked to the surfaces of magnetic beads. | 1.75×10^3^ - 7.0×10^6^ particles/μL | 1.16 × 10^3^ particles/µL | S11 |

**Table S2.** Clinical characteristics of patient samples.

| **Characteristic** | **Healthy**  **(*n* = 10)** | **Benign**  **(*n* = 10)** | **Ovarian cancer**  **(*n* = 10)** |
| --- | --- | --- | --- |
| Age (years) | 56 ± 11 (45-72) | 62 ± 71 (39-71) | 62 ± 12 (39-70) |
| CA-125 (U/mL) | (-) | (-) | 893 ± 760 (33-2020) |
| Pathologic Diagnosis | (-) | (-) | High grade serous 10/10 |
| Clinical stage | (-) | (-) | IIIC (10/10) |
| Primary or recurrence | (-) | (-) | Primary (10/10) |
| Optimally debulked | (-) | (-) | 10/10 |

**Data are presented as mean ± SD (min-max). (−) Data not applicable.*

**References**

1. Salomon, C.; Kobayashi, M.; Ashman, K.; Sobrevia, L.; Mitchell, M. D.; Rice, G. E. *PLoS One* **2013,** *8*, e79636.
2. Rice, G. E.; Scholz-Romero, K.; Sweeney, E.; Peiris, H.; Kobayashi, M.; Duncombe, G.; Mitchell, M. D.; Salomon, C. *J. Clin. Endocrinol. Metab.* **2015**, *100*, E1280-8.
3. Weisberg, S. P.; McCann, D.; Desai, M.; Rosenbaum, M.; Leibel, R. L.; Ferrante, A. W., Jr. *J. Clin. Invest.* **2003**, *112*, 1796-808.
4. Weisberg, S. P.; Hunter, D.; Huber, R.; Lemieux, J.; Slaymaker, S.; Vaddi, K.; Charo, I.; Leibel, R. L.; Ferrante, A. W., Jr. *J. Clin. Invest.* **2006**, *116*, 115-24.
5. Farhana, F. Z.; Umer, M.; Saeed, A.; Pannu, A. S.; Shahbazi, M.; Jabur, A.; Nam, H. J.; Ostrikov, K.; Sonar, P.; Firoz, S. H. *ACS Appl. Nano Mater*. **2021**, *4* (2), 1175-1186.
6. Tian, Y.-F.; Ning, C.-F.; He, F.; Yin, B.-C.; Ye, B.-C. *Analyst* **2018**, 143 (20), 4915-4922.
7. Wang, C.; Huang, C.-H.; Gao, Z.; Shen, J.; He, J.; MacLachlan, A.; Ma, C.; Chang, Y.; Yang, W.; Cai, Y. *ACS Sens*ors **2021**, *6* (9), 3308-3319.
8. Wang, Z.; Zong, S.; Wang, Y.; Li, N.; Li, L.; Lu, J.; Wang, Z.; Chen, B.; Cui, Y. *Nanoscale* **2018**, *10* (19), 9053-9062.
9. Wang, Q.; Zou, L.; Yang, X.; Liu, X.; Nie, W.; Zheng, Y.; Cheng, Q.; Wang, K. *Biosens. Bioelectron*. **2019**, *135*, 129-136.
10. Li, P.; Yu, X.; Han, W.; Kong, Y.; Bao, W.; Zhang, J.; Zhang, W.; Gu, Y. *ACS Sensors* **2019**, *4* (5), 1433-1441.
11. Gao, M.-L.; He, F.; Yin, B.-C.; Ye, B.-C. *Analyst* **2019**, *144* (6), 1995-2002.
